# Association between Cognitive Impairment and Malnutrition in Hemodialysis Patients: Two Sides of the Same Coin

**DOI:** 10.3390/nu15040813

**Published:** 2023-02-04

**Authors:** Silverio Rotondi, Lida Tartaglione, Marzia Pasquali, Maria Josè Ceravolo, Anna Paola Mitterhofer, Annalisa Noce, Monica Tavilla, Silvia Lai, Francesca Tinti, Maria Luisa Muci, Alessio Farcomeni, Sandro Mazzaferro

**Affiliations:** 1 Nephrology and Dialysis Unit, ICOT Hospital, Polo Pontino Sapienza University of Rome, 04100 Rome, Italy; 2 Department of Translational and Precision Medicine, Policlinico Umberto I Hospital, Sapienza University of Rome, Viale del Policlinico 155, 00161 Rome, Italy; 3 Nephrology Unit, Department of Internal Medicine and Medical Specialities, University Policlinico Umberto I Hospital, 00161 Rome, Italy; 4 Nephrology and Dialysis Unit, University Hospital Policlinico Tor Vergata, 00133 Rome, Italy; 5 Department of Systems Medicine, University Hospital Policlinico Tor Vergata, 00133 Rome, Italy; 6 Nephrology an Dialysis Unit, Fatebenefratelli Isola Tiberina Fondazione Policlinico Universitario A. Gemelli-Isola, 00186 Rome, Italy; 7 Department of Economics & Finance, University of Rome “Tor Vergata”, 00133 Rome, Italy

**Keywords:** hemodialysis, protein energy wasting, mild cognitive impairment, MIS, MoCa

## Abstract

Cognitive impairment and malnutrition are prevalent in patients on hemodialysis (HD), and they negatively affect the outcomes of HD patients. Evidence suggests that cognitive impairment and malnutrition may be associated, but clinical studies to assess this association in HD patients are lacking. The aim of this study was to evaluate the association between cognitive impairment evaluated by the Montreal Cognitive Assessment (MoCA) score and nutritional status evaluated by the malnutrition inflammation score (MIS) in HD patients. We enrolled 84 HD patients (44 males and 40 females; age: 75.8 years (63.5–82.7); HD vintage: 46.0 months (22.1–66.9)). The MISs identified 34 patients (40%) as malnourished; the MoCa scores identified 67 patients (80%) with mild cognitive impairment (MCI). Malnourished patients had a higher prevalence of MCI compared to well-nourished patients (85% vs. 70%; *p* = 0.014). MoCa score and MIS were negatively correlated (rho:−0.317; *p* < 0.01). Our data showed a high prevalence of MCI and malnutrition in HD patients. Low MoCA scores characterized patients with high MISs, and malnutrition was a risk factor for MCI. In conclusion, it is plausible that MCI and malnutrition are linked by common sociodemographic, clinical, and biochemical risk factors rather than by a pathophysiological mechanism.

## 1. Introduction

Cognitive impairment ranges from mild (MCI; minor or moderate cognitive impairment which does not interfere with independence in daily activities) to severe impairment (major cognitive impairment or dementia, usually involving at least two domains and interfering with independence in everyday activities). Chronic kidney disease (CKD) is one of the strongest risk factors for MCI and dementia [1], with evidence of cognitive impairment in the early stages of CKD. The reported prevalence of cognitive impairment in CKD is highly variable (10% to 60%) and is influenced by the CKD stage and the method used for cognitive impairment assessment [2,3]. In hemodialysis (HD) patients, cognitive impairment is extremely common, and more than 70% of patients show impairment in at least one cognitive domain [4]. The causes of CKD-related cognitive impairment are varied and include both traditional cardiovascular (CV) risk factors (diabetes, hypertension, and dyslipidemia) and nontraditional kidney-disease-related factors. Nontraditional risk factors for cognitive impairment include uremic toxins, hemodialysis factors (rapid fluid shift, hypotension, etc.), inflammation, and comorbidities such as depression and anemia [5,6].

In addition, malnutrition is highly prevalent in HD patients. In this population, nutritional and metabolic alterations often coexist and result in a progressive loss of body stores of protein and energy fuels (i.e., body muscle and fat mass), leading to protein-energy wasting (PEW) [7]. A diagnosis of PEW can be associated with negative outcomes for a patient’s prognosis and quality of life, as well as health economic challenges. PEW prevalence in CKD, as assessed using a subjective global assessment (SGA) or malnutrition inflammation score (MIS), is about 40% or higher [8]. The causes of malnutrition or, rather, protein-energy wasting (PEW) are multifactorial and include iatrogenic and noniatrogenic factors. Among iatrogenic causes, the most common are dialysis-induced nutrient losses, dialysis-induced inflammation, the efficacy of uremia correction, dialysis adequacy and frequency, and the efficacy of metabolic acidosis correction. Noniatrogenic causes include suboptimal dietary intake, poor appetite, depression, lack of social support, and decreased physical activity.

A lack of specific nutrients that are essential for neurotransmission [9], such as vitamin D and magnesium, rather than the over-inflammation and increased oxidative stress observed in malnutrition [10], can contribute to cognitive impairment in HD patients. Furthermore, both cognitive impairment and malnutrition are influenced by similar risk factors (depression, inflammation, anemia, and metabolic acidosis).

Only a few studies have evaluated the association between nutritional status and cognitive symptoms in patients affected by CKD. However, previous studies have enrolled patients on conservative therapy affected by advanced CKD [11] or patients on peritoneal dialysis with end-stage renal disease [12]. For hemodialysis patients, available studies are limited [13,14]. In fact, despite the guidelines on nutrition in CKD and some evidence emphasizing the importance of nutritionists and psychologists in hemodialysis centers, these specialists are rarely present. [15,16]. This renders nutritional and cognitive assessments difficult to obtain [17]. Moreover, to the best of our knowledge, the only multicentre study in the literature evaluating cognitive and nutritional status in hemodialysis patients comes from a southwestern Chinese population, which makes the results country-specific [18].

The purpose of the present study was to investigate whether cognitive impairment is associated with nutritional status in HD patients.

## 2. Research Design and Methods

### 2.1. Study Design and Data Collection

This was an epidemiological survey recruiting 84 patients (40 women and 44 men) with end-stage renal disease undergoing hemodialysis at the Dialysis and Nephrology Unit of Marco Pasquali Institute I.C.O.T, Polo Pontino Sapienza University of Rome, between January and December 2021. The inclusion criteria were age ≥18 years and chronic HD treatment for at least three months. The exclusion criterion were: evidence of major cognitive impairment or dementia according to the DSM-5 (Diagnostic and Statistical Manual for Mental Disorders) diagnosed by the psychiatrist or geriatrician; presence of a condition that could independently influence nutritional status (active cancer, decompensated chronic liver disease, severe heart failure, or intestinal malabsorption); and concomitant therapy with medication that could alter cognitive evaluation as opioids, antidepressants, benzodiazepines, lithium, antiparkinsonians drugs. This study was a subanalysis of protocol N° 0027769/2019 “https://www.aslroma2.it/COMITATOETICO/” (accessed on 8 November 2018), which was approved in 2017 by the “Comitato Etico Lazio 2” EC. The study was conducted in accordance with the Declaration of Helsinki and received informed consent approval from all involved patients.

The following data were collected upon enrollment: 

Sociodemographic variables: age, sex, and level of education;

Clinical data: comorbidities (diabetes mellitus, hypertension, and history of cardiovascular events), dialysis vintage, and systolic and diastolic blood pressure (pre- and post-HD session), chronic drug therapy.

### 2.2. Nutritional Evaluation

Body mass index (BMI), malnutrition inflammation score (MIS), and protein and energy intake were obtained from a 3-day food record. The MIS is currently considered a suitable tool to assess the nutritional status of patients with severe CKD (both predialytic and dialytic) [14], and it was correlated with the morbidity and mortality of these subjects [19]. It includes four sections: (1) medical history (change in dry weight, dietary intake, gastrointestinal symptoms, functional capacity, and comorbidities), (2) physical examination (decreased fat stores or loss of subcutaneous fat and signs of muscle wasting), (3) BMI, and (4) laboratory parameters (serum albumin and total iron-binding capacity). Each item is scored from 0 to 3, and the total score can range from 0 (normal) to 30 (severely malnourished). An MIS ≥8 identifies patients affected by malnutrition [20].

### 2.3. Cognitive Evaluation

Cognitive functions were evaluated by a dedicated psychologist using the Montreal Cognitive Assessment (MoCA), corrected for education. The MoCA is a brief cognitive screening test exploring eight cognitive domains (visuospatial/executive, naming, memory, attention, language, abstraction, delayed recall, and orientation), with high sensitivity and specificity for detecting the earliest stages of cognitive decline at any level of education [21]. The MoCA was administered in the week after enrollment and before the first HD session (after a long interdialytic interval). We used the accepted cut-off of 26 to define cognitive impairment [21].

### 2.4. Laboratory Data

Blood samples were collected before and after the same HD session at which the MoCA was performed for the assessment of hemoglobin (Hb), calcium (Ca), phosphate (P), magnesium, parathyroid hormone (PTH), calcifediol (25D), albumin, C-reactive protein (CRP, vn 0–0.5 mg/dL), total cholesterol (predialysis), urea, and bicarbonatemia (predialysis and postdialysis). iPTH was assayed with a chemiluminescent microparticle immunoassay (CMIA) based on a two-step sandwich immunoassay for the quantitative determination of intact parathyroid hormone (Architect system, Abbott Diagnostic, Wiesbaden, Germany). Normal values were 20–104 pg/mL, with a measurement range from 3 to 3000 pg/mL.

### 2.5. Statistical Analysis 

Data are expressed as means ± standard deviation (SD) for Gaussian variables or medians [25th–75th percentiles] when normality was not tenable. We used the Shapiro test to evaluate the normality of continuous measurements. Chi-squared tests were used for qualitative variables. T-tests were used to compare measurements between groups for quantitative variables. When a normality assumption was not tenable, Mann–Whitney tests were used to test for any significant differences. 

Spearman’s correlation was used to assess the monotonic covariation of measurements. Multivariate regression analysis was applied to estimate the predictive parameters of MCI and MIS, as well as to account for the roles of the different variables considered. The final multivariate model was obtained by minimizing the Akaike information criterion via a backward stepwise regression. All tests were two-tailed and (adjusted) *p*-values < 0.05 were considered statistically significant.

Analyses were performed using R open-source software, version 3.4.0. R Foundation for Statistical Computing, Vienna, Austria.

## 3. Results

Eighty-four patients (44 males and 40 females) with a median age of 75.8 (63.5–82.7) years and on replacement therapy for an average of 46.0 (22.1–66.9) months fulfilled the inclusion criteria and consented to be enrolled in the study. Six patients were excluded: 4 for the presence of major cognitive impairment or dementia and 2 refused to give informed consent. Hemodialysis treatment was received via a central venous catheter (CVC) in 26% of the patients and via arteriovenous fistula in 74% of the patients. Regarding comorbidities, arterial hypertension was present in 72 of the patients (86%), with cardiovascular disease in 35 patients (42%) and diabetes mellitus in 22 patients (26%). An analysis of education level showed that 29 patients (34.5%) had more than 8 years of education, and 9.5% of the sample had no formal education. As for biochemical parameters, calcium (9.01 ± 0.59 mg/dL), magnesium (1.91 ± 0.85 mEq/L) and PTH (325.0 [194.5–496.7 pg/mL]) were within the recommended ranges, but patients showed increased levels of phosphate (5.16 ± 1.30 mg/dL) and low levels of 25D (20.3 ± 7.0 ng/mL). In addition, hemoglobin was within the recommended range (10,71 ± 1,12 g/dL), and there was evidence of mild inflammation (CRP = 0.98 ± 1.86 mg/dL). Regarding medication, 86% (*n*. 72) of patients were on antihypertensive therapies, 26% (*n*. 22) on insulin therapy and 20% (*n*. 16) on nutritional Vitamin D therapy. Descriptive analyses of the total samples are reported in Table 1. 

The mean body mass index showed a tendency toward overweight values (25.9 ± 6.4). The mean albumin levels were in the normal range (3.73 ± 0.38 gr/dL). An analysis of 3-day food records showed a protein intake of 1.04 ± 0.21 gr/Kg/day and an energy intake of 24.92 ± 5.24 Kcal/Kg/day (Table 1).

Malnutrition, as evaluated according to the prespecified MIS threshold, was present in 40% of the sample patients (*n* = 34).

The mean MoCA scores were pathological (21.2 ± 4.7) and identified mild cognitive impairment in 67 patients (80%). Only 20% of the sample (*n* = 17) had no cognitive impairment (Table 1).

**Figure 1:**
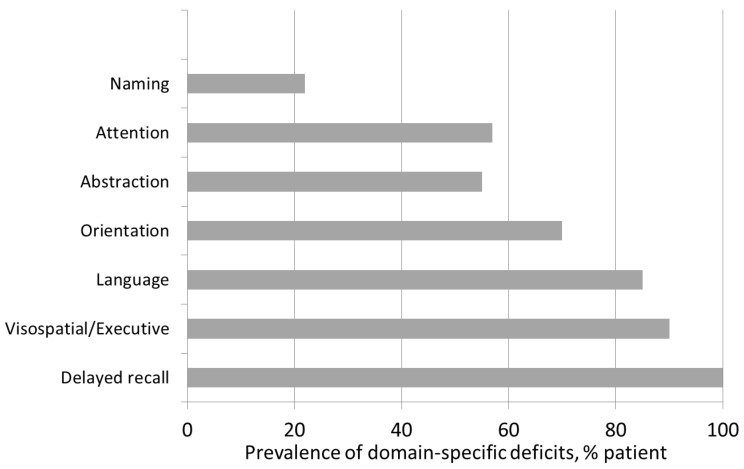
Prevalence of domain-specific deficits in HD patients.

When analyzing the specific cognitive domains within the MoCA, memory deficit was the most frequent impairment (100%), followed by visuospatial/executive dysfunction (90%) and language deficit (85%). Moderate and severe impairments were also found in the domains of abstraction (55%), attention (30%), and orientation (30%) (Figure 1).

We analyzed the main clinical and biochemical parameters between the malnourished and well-nourished subgroups (Table 2), as well as between the subgroups with and without cognitive impairment (Table 3).

Malnourished patients were older (79.8 [71.2–84.6] vs. 61.5 [47.4–70.0] years; *p* = 0.019), were on dialysis for longer times (56.0 [39.0–76.9] vs. 33.0 [20.0–59.0] months; *p* < 0.018) and had lower levels of education (years of education <8:55.9 vs. 26.0%; *p* = 0.007). Postdialytic blood pressure was lower in malnourished patients (69.1 ± 9.5 vs. 74.7 ± 9.8; *p* = 0.003). Among other biochemical parameters, albumin was lower (3.64 ± 0.36 vs. 3.80 ± 0.36 gr/dL; *p* = 0.049), while CRP (1.68 ± 2.89 vs. 0.55 ± 0.38 mg/dL; *p* = 0.024) and parathyroid hormone (513.92 ± 432.14 vs. 325.35 ± 243.61 pg/mL; *p* = 0.016) were higher in malnourished patients. We did not observe any differences in BMI (26.0 ± 6.5 vs. 26.1 ± 6.6) or protein (1.09 ± 0.24 vs. 1.01 ± 0.19) and energy (25.7 ± 5.4 vs. 24.3± 5.2) intakes between the malnourished and well-nourished subgroups (Table 2). No differences in antihypertensive (85%; *n* = 29 vs. 86%; *n* = 43; *p*: 0.999), insulin (35%; *n*= 12 vs. 20%; *n* = 10; *p*: 0.189) and nutritional vitamin D (21%; *n* = 7 vs. 19%; *n* = 9; *p*: 0.998) therapies were found between malnourished and well-nourished groups, respectively.

The MIS was correlated positively with age (rho = −0.309; *p* = 0.004) and CRP (rho = 0.339; *p* = *0*.003) and negatively with albumin (rho = −0.300; *p* = 0.005) and postdialytic diastolic blood pressure (rho = −0.229; *p* = 0.035).

A multivariate analysis showed only PTH (OR: 1.0026; 95% CI: 1.0009–1.0049; *p* = 0.009) and age (OR: 1.0537; 95% CI: 1.0133–1.1034; *p* = 0.014) as independent positive predictors of malnourishment. 

**Figure 2:**
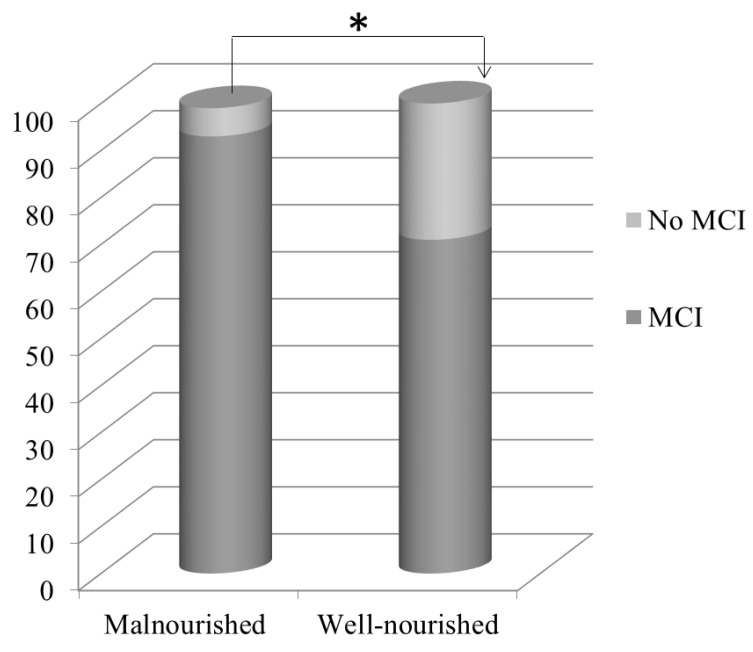
Prevalence of MCI in malnourished and well-nourished patients. Malnourished patients showed a higher percentage of MCI (85%) compared to well-nourished patients (70%). (*) 85% vs. 70%; *p* = 0.014.

Malnourished patients showed a higher percentage of MCI (85%; *n* = 29/34) compared to well-nourished patients, of which 70% (*n* = 35/50) showed cognitive impairment (*p* = *0*.014; Figure 2). 

Patients with MCI were older (78.7; 72.1–84.5 vs. 61.5; 47.4–70.0 years; *p* < 0.001) and had lower levels of education (years of education <8:46.3 vs. 11.7%; *p* = 0.011) than patients without cognitive impairment. Diabetes was more frequent in patients with MCI (46.3 vs. 11.7%; *p* = 0.034). Predialysis diastolic arterial blood pressure was lower in the MCI subgroup (69.6 ± 10.3 vs. 77.0± 8.1; *p* = 0.0006). No differences in antihypertensive (83%; *n* = 56 vs. 94%; *n* = 16; *p*: 0.275) and nutritional vitamin D (18%; *n* = 12 vs. 22%; *n* = 4; *p*:0.890) therapies were found between MCI and no-MCI groups, respectively. Instead, insulin therapy was more prevalent in the MCI group (31%; *n* = 21 vs. 6%; *n* = 1; *p*:0.034). Table 3 shows a comparison of the clinical and biochemical features between these two subgroups.

**Figure 3:**
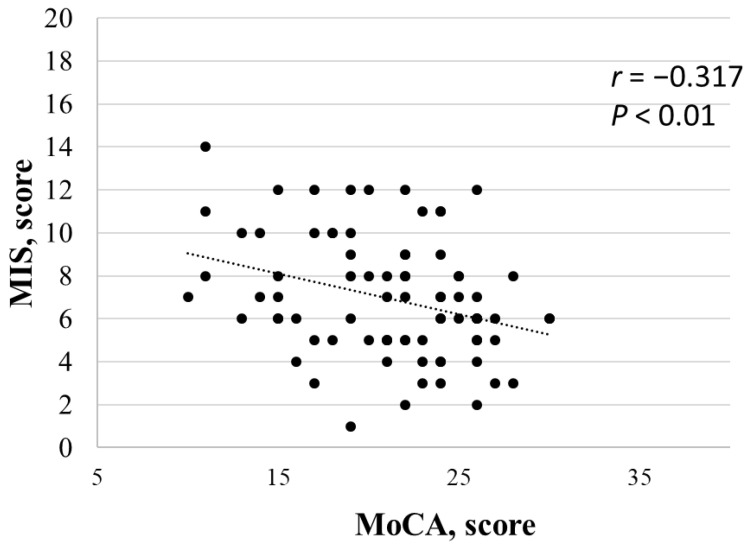
Correlation test between MIS and MoCA score. Spearman’s correlation was used to assess the monotonic covariation of measurements.

MoCA score correlated negatively with age (rho = −0.580; *p* < 0.001) and positively with predialytic (rho = 0.376; *p* = <0.001) and postdialytic (rho = 226; *p* < 0.050) diastolic blood pressures. The multivariate analysis confirmed only age as an independent predictor of cognitive impairment (*p* < 0.01). Further, the mean MoCA scores were lower in malnourished patients than in well-nourished patients (19.5 ± 4.6 vs. 22.1 ± 4.6; *p* = 0.046). Lastly, the MIS and MoCA scores were negatively correlated (rho = −0.371; *p* = 0.004; Figure 3).

## 4. Discussion

Our study showed a high prevalence of mild cognitive impairment (80%) and malnutrition (40%) in the studied HD population. Malnutrition and MCI results revealed a higher prevalence of MCI in the malnourished group compared to the well-nourished group (Figure 2) and a negative correlation between the MIS and MoCA score (Figure 3). 

### 4.1. Nutritional Evaluation

We observed that 40% of the patients were malnourished, in line with previous larger clinical studies. In addition, we observed inadequate energy and protein intakes (24.92 ± 5.24 Kcal/Kg/day and 1.04 ± 0.21 gr/Kg/day, respectively) below the thresholds recommend by more recent DKOQI guidelines (25–35 Kcal/Kg/day and 1.0–1.2 g/Kg/day, respectively) [14].

However, these recommended quantities are difficult to reach for HD patients due to limitations represented by both dietary restrictions and socioeconomic conditions. 

In our study, the main factors affecting nutritional status were age, dialysis vintage, albumin level, inflammation, blood pressure, and secondary hyperparathyroidism. 

Aging is a classic risk factor for malnutrition due to reduced intake of nutrients (particularly protein), increased anorexia, and increased comorbidities. In our population, malnourished patients were older than well-nourished patients, and this association was also confirmed by univariate and multivariate analyses. 

In addition, the association with albumin was not surprising. Albumin level is a sensitive method for identifying patients at risk for PEW [22], and an albumin value < 3.8 g/dL is a biochemical marker for the diagnosis of protein–energy alteration (together with transthyretin and cholesterol) [23]. However, albumin can also reflect many non-nutritional factors that frequently occur among CKD patients, including anemia, infections, urinary and dialysate losses, and hydration status [24]. In our HD patients, we found that mean albumin levels were lower in malnourished patients (3.6 gr/dL) than in well-nourished patients, and we also observed a positive correlation between albumin levels and MIS in the univariate analysis. On the other hand, malnourished patients also had higher inflammation, as shown by CRP levels. Inflammation is a well-known risk factor for PEW development [25], and the association between these two conditions is also known as malnutrition–inflammation cachexia syndrome (MICS) [26]. Moreover, PTH levels were higher in malnourished patients and were correlated positively with MIS in both the univariate and multivariate analyses. Hyperparathyroidism in a clinical setting can lead to an increase in resting energy expenditure and muscle wasting [27,28,29], and clinical and experimental studies have suggested that excess PTH leads to adipose tissue browning, explaining the reduction in muscle mass [30,31]. We can guess a direct role of PTH in muscle wasting in our population. We also found a vitamin D insufficiency in the whole population. However, despite the role of vitamin D in neurotransmitter synthesis, nerve growth and cognitive functions, we did not observe its involvement in cognitive impairment of our patients.

Regarding blood pressure, we observed a correlation between postdialytic blood pressure and MIS. We supposed that lower DBP levels could be indicative of reduced organ perfusion and orthostatic hypotension, both risk factors for malnutrition or the risk of malnutrition [32,33]. 

### 4.2. Cognitive Evaluation

Previous clinical studies have reported cognitive impairment to be present in 10–40% of chronic kidney disease patients. Murray et al. observed that, in a large cohort of HD patients, only 13% of the patients had normal cognitive function, while 50% had mild to moderate cognitive impairment and 37% had severe cognitive impairment [34]. A multicenter study of Italian HD patients also showed that only 28.9% of the patients were not impaired in any cognitive domain [4]. In our population, MCI was evident in more than half of the sample (80%), in line with recent data. In previous studies, cognitive impairment has been associated with age [35], a low level of education (<8 years of education) [34], and specific clinical conditions and comorbidities (depression, cardiovascular disease, stroke, and anemia [6]), as well as with dialytic factors (Kt/V [35], hemodynamic changes associated with hemodialysis [36], and inflammation). In our study, the main factors affecting MCI were age, level of education, diabetes, and diastolic blood pressure (DBP). Both age and diabetes are well-known risk factors for cognitive impairment not only in CKD patients, but also in the general population [37]. Elderly individuals with diabetes can develop small-vessel cerebrovascular disease, an important factor in the development of CKD-related cognitive impairment [38,39]. Furthermore, elderly individuals are at risk for developing deficits in the memory cognitive domain [6]. Notably, in our population, memory and executive function were the cognitive domains most affected (Figure 1). 

Memory storage capacity is notoriously impaired in patients with CKD, in whom both implicit (procedural, unconscious) and explicit (declarative, conscious) forms of memory seem to be altered [40]. It is well-documented that explicit memory declines with normal aging [41], but it is less clear whether implicit memory declines or remains stable with normal aging. In conclusion, aging seems to be responsible for memory dysfunction in CKD, but it is not the only factor involved. A recent study showed that characteristic modifications of neuronal synapses regarding cholinergic neurons could be responsible for memory deficits in CKD patients [42].

In parallel, cerebrovascular disease is significantly associated with deficits in executive function. Several studies have demonstrated that processing speed and executive function are the domains most affected in individuals with CKD [38]. In our population, 26% (*n* = 22) of the patients had diabetes, and almost all of these (*n* = 21) displayed MCI, underlining the importance of diabetes in cognitive dysfunction. Furthermore, 42% had CVD, and 86% had arterial hypertension. Alterations in executive domain function were, therefore, expected. 

Despite the high prevalence of arterial hypertension, low DBP seemed to be related to MCI. In the general population, reduced cerebral blood flow impairs neural activity, which is involved in the pathogenesis of cognitive impairment. In hemodialysis, rapid fluid shifts can often lead to wide swings in blood pressure, intradialytic hypotension, and brain injury with cerebral atrophy [43]. Cerebral blood flow declines significantly during dialysis and greater decreases in cerebral blood flow are associated with worse cognitive function [36]. We did not measure cerebral blood flow, but we observed a relationship between MoCA score and diastolic blood pressure. 

Diastolic blood pressure (DBP), the steady component of blood pressure, can reflect vasoconstriction and cerebral perfusion pressure. In a previous study, DBP was associated with risk of dementia in elderly individuals [44]. In our study, patients with MCI had predialysis DBPs lower than patients without cognitive impairment. In addition, postdialytic DBP seemed to be lower in MCI patients (70.8 ± 10.0 vs. 75.9 ± 9.8), although the difference did not reach the level of significance. Indeed, both pre- and postdialysis DBPs were correlated with MoCA score in the univariate analysis. We also found vitamin D insufficiency in the whole population. However, despite the role of vitamin D in neurotransmitter synthesis, nerve growth and cognitive functions, we had no evidence of its possible involvement in cognitive impairment. 

Finally, we observed that MCI and malnutrition were associated. Indeed, despite completely different items in their score, the MoCA score and MIS correlated, and the prevalence of MCI was higher in malnourished patients. However, the multivariate analysis did not confirm MCI as an independent predictor of nutritional status. It is therefore possible that MCI and malnutrition were linked by common risk factors (age, blood pressure, and comorbidities) responsible for the high prevalence of both observed in our population.

### 4.3. Limitations of the Study

However, this study had some limitations. First, this was an epidemiological survey in which a limited number of subjects was recruited. A larger-scale prospective study is required to confirm the risk factors for developing MCI and malnutrition. Second, our study lacked a group of healthy controls necessary to estimate the strength of the association between malnutrition and cognitive impairment in CKD. Third, we administered the MoCA test for the diagnosis of MCI (currently considered an appropriate tool for screening cognitive impairment), but we did not administer more accurate neuropsychological tests that were specific to each cognitive domain. However, at the moment, there are few reports in the literature of studies of malnutrition and cognitive impairment in HD patients. Fourth, the relatively old age of our patients could be a determinant of the obtained results. However, the average age of our patients is similar to that of the dialysis population in Europe [45,46,47]. Thus, our population can be regarded as representative of a typical HD population. In addition, we observed a higher prevalence of MCI and malnutrition compared to an age-matched population without CKD [48,49]. This identifies the role of CKD in increasing the risk of nutritional and cognitive alterations [50]. Our results suggest a link between these two aspects, implying the need to evaluate them to decide on combined nutritional–cognitive therapeutic interventions to improve patient outcomes.

## 5. Conclusions

We observed a high prevalence of both MCI and malnutrition in our HD patients. In malnourished patients, we further observed a higher prevalence of MCI. The main factors involved were sociodemographic (age and level of education), clinical (diastolic blood pressure), and biochemical (secondary hyperparathyroidism) factors. The multivariate analysis confirmed age as an independent variable for both MCI and malnutrition. Notably, PTH level was an independent predictor of malnutrition. 

It is plausible that MCI and malnutrition are linked by common risk factors rather than by a pathophysiological mechanism.

## Figures and Tables

**Table 1:** Clinical and biochemical characteristics.

n	84
Age, years	75.8 (63.5–82.7)
Sex, M/F (%)	44/40 (52/48)
BMI, Kg/m^2^	25.9 ± 6.4
Dialysis vintage, months	46.0 (22.1–66.9)
Vascular access, n (%) - CVC - AVF	22 (26) 62 (74)
Level of education, n (%) - <5 years - ≤8 years - >8 years	8 (9.5) 47 (55.5) 29 (34.5)
Comorbidities, n (%) - Arterial hypertension - Diabetes mellitus - Cardiovascular disease	72 (86) 22 (26) 35 (42)
Pre-HD SBP, mmHg	134.3 ± 17.8
Post-HD SBP, mmHg	133.1 ± 16.2
Pre-HD DBP, mmHg	71.5 ± 11.6
Post-HD DBP, mmHg	71.8 ± 10.4
Calcium, mg/dL	9.01 ± 0.59
Phosphate, mg/dL	5.16 ± 1.30
Magnesium, meq/L	1.91 ± 0.85
25D, ng/mL	20.3 ± 7.0
Uric acid, mg/dL	5.9 ± 1.0
PTH, pg/mL	325.0 (194.5–496.7)
Hb, gr/dL	10.7 ± 1.1
CRP, mg/dL	0.98 ± 1.86
KT/V	1.43 ± 0.24
Pre-HD bicarbonate, mmol/L	21.1 ± 2.8
MoCA, score	21.1 ± 2.8
MCI, n (%)	67 (80)
Malnourishment, n (%)	34 (40)
Protein intake, gr/Kg/day	1.04 ± 0.24
Energy intake, Kcal/Kg/day	24.9 ± 5.24

Abbreviations: M—male; F—female; CVC—central venous catheter; AVF—arteriovenous fistula; SBP—systolic blood pressure; DBP—diastolic blood pressure; 25D—calcifediol; PTH—parathyroid hormone; Hb—hemoglobin; CRP—C-reactive protein; MCI— mild cognitive impairment.

**Table 2:** Clinical and biochemical characteristics of HD patients according to nutritional status.

	Malnourished (*n* = 34)	Well-Nourished (*n* = 50)	*p*-Value
Age, years	79.8 (71.2–84.6)	61.5 (47.4–70.0)	0.011
Sex, M/F (%)	15/19 (44.1/55.9)	29/21 (58.0/42.0)	0.304
Dialysis vintage, months	56.0 (39.0–76.9)	33.0 (20.0–59.0)	0.018
Vascular access, *n* (%) - CVC - AVF	9 (26.5) 25 (73.5)	13 (26.0) 37 (74.0)	0.999
Levels of education, *n* (%) - ≤8 years - >8 years	19 (55.9) 15 (44.1)	13 (26.0) 37 (74.0)	0.006
Diabetes mellitus, *n* (%) - No - Yes	22 (64.7) 12 (35.3)	40 (80.0) 10 (20.0)	0.189
Arterial hypertension, *n* (%) - No - Yes	5 (14.7) 29 (85.3)	7 (14.0) 43 (86.0)	0.999
Cardiovascular disease, *n* (%) - No - Yes	19 (55.9) 15 (44.1)	30 (60.0) 20 (40.0)	0.880
Pre-HD SBP, mmHg	135.6 ± 19.3	134.5 ± 16.4	0.773
Post-HD SBP, mmHg	133.8 ± 17.3	132.9 ± 15.5	0.705
Pre-HD DBP, mmHg	70.2 ± 11.6	73.6 ± 10.4	0.072
Post-HD DBP, mmHg	69.1 ± 9.5	74.7 ± 9.8	0.023
Calcium, mg/dL	9.0 ± 0.6	9.0 ± 0.6	0.910
Phosphate, mg/dL	5.3 ± 1.5	5.0 ± 1.2	0.407
Magnesium, meq/L	2.01 ± 0.61	1.90 ± 0.91	0.970
25D, ng/mL	21.5 ± 6.2	19.6 ± 8.2	0.072
Uric acid, mg/dL	5.8 ± 1.0	6.0 ± 1.1	0.556
PTH, pg/mL	361.0 (302.3–607.3)	245.5 (177.3–407.8)	0.014
Hb, gr/dL	10.9 ± 1.0	10.6 ± 1.1	0.260
CRP, mg/dL	1.68 ± 2.89	0.55 ± 0.38	0.002
KT/V	1.48 ± 0.22	1.49 ± 0.26	0.133
Pre-HD bicarbonate, mmol/L	21.8 ± 2.8	20.5 ± 2.8	0.065
Albumin, gr/dL	3.6 ± 0.3	3.8 ± 0.3	0.049
MCI, *n* (%)	29 (85)	35 (70)	0.014
MoCA, score	19.5 ± 4.6	22.1 ± 4.6	0.046

Malnourished patients were identified by an MIS ≥ 8. Quantitative data are shown as means ± standard deviation or medians (IQR) according to normality. Qualitative data are shown as numbers (percentage). Chi-squared tests were used for qualitative variables. T-tests or Mann–Whitney tests were used to compare measurements between malnourished and well-nourished subgroups according to normality. Abbreviations: M—male; F—female; CVC—central venous catheter; AVF—arteriovenous fistula; SBP—systolic blood pressure; DBP—diastolic blood pressure; 25D—calcifediol; PTH—parathyroid hormone; Hb—hemoglobin; CRP—C-reactive protein.

**Table 3:** Clinical and biochemical characteristics of HD patients according to MCI diagnosis.

	MCI (*n* = 67)	No MCI (*n* = 17)	*p*-Value
Age, years	78.7 (72.1–84.5)	61.5 (47.4–70.0)	<0.0001
Sex, M/F (%)	33/34; (49.2/50.8)	10/7 (58.8/41.2)	0.664
Dialysis vintage, months	46.0 (24.3–69.0)	46.0 (19.0–59.0)	0.470
Vascular access, *n* (%) - CVC - AVF	16 (23.9) 51 (76.1)	7 (41.2) 10(58.8)	0.261
Level of education, *n* (%) - ≤8 years - >8 years	31 (46.3) 36 (53.7)	2 (11.7) 15 (88.3)	0.011
Diabetes mellitus, *n* (%) - No - Yes	46 (68.7) 21 (31.3)	16 (94.1) 1 (5.9)	0.034
Arterial hypertension, *n* (%) - No - Yes	11 (16.4) 56 (83.5)	1 (5.9) 16 (94.1)	0.275
Cardiovascular disease, *n* (%) - No - Yes	40 (59.7) 27 (40.3)	11 (64.7) 6 (35.3)	0.920
Pre-HD SBP, mmHg	134.1 ± 18.2	135.7 ± 12.3	0.873
Post-HD SBP, mmHg	133.4 ± 15.9	132.2 ± 17.5	0.876
Pre-HD DBP, mmHg	69.6 ± 10.3	78.0 ± 8.1	0.006
Post-HD DBP, mmHg	70.8 ± 10.0	75.9 ± 9.8	0.069
Calcium, mg/dL	9.1 ± 0.6	8.7 ± 0.7	0.060
Phosphate, mg/dL	5.2 ± 1.4	5.3 ± 1.2	0.691
Magnesium, meq/L	1.92 ± 0.6	1.91 ± 0.91	0.930
Uric acid, mg/dL	5.9 ± 0.9	6.0 ± 1.5	0.839
PTH, pg/mL	325.0 (195.7–508.2)	350.0 (213.0–413.0)	0.930
25D, ng/mL	20.6 ± 6.4	20.1 ± 7.5	0.332
Hb, gr/dL	10.7 ± 1.1	10.5 ± 1.3	0.839
CRP, mg/dL	0.79 ± 1.02	0.51 ± 0.29	0.361
KT/V	1.45 ± 0.21	1.42 ± 0.34	0.552
Pre-HD bicarbonate, mmol/L	21.0 ± 2.7	21.0 ± 3.3	0.548
Albumin, gr/dL	3.7 ± 0.4	3.8 ± 0.3	0.303

MCI was identified by a MoCA score < 26. Quantitative data are shown as means ± standard deviation or medians (IQR) according to normality. Qualitative data are shown as numbers (percentage). Chi-squared tests were used for qualitative variables. T-tests or Mann–Whitney tests were used to compare measurements between the MCI and no-MCI subgroups, according to normality. Abbreviations: M—male; F—female; CVC—central venous catheter; AVF—arteriovenous fistula; SBP—systolic blood pressure; DBP—diastolic blood pressure; 25D—calcifediol; PTH—parathyroid hormone; Hb—hemoglobin; CRP—C-reactive protein.

## Data Availability

Correspondence and requests for materials should be addressed to L.T.
